# Performance of Marine Anammox *Candidatus* Scalindua sp. under High Nitrate Conditions in a Biofilm Reactor

**DOI:** 10.1264/jsme2.ME25094

**Published:** 2026-05-27

**Authors:** Jonathan A.C. Roques, Naoki Fujii, Ebuka Unegbu, Amélie Marqué, Emma Johansson, Kohei Yamamoto, Haruhi Iida, Tomonori Kindaichi

**Affiliations:** 1 Department of Biological and Environmental Sciences, University of Gothenburg, Box 463, 405 30, Gothenburg, Sweden; 2 Swedish Mariculture Research Center (SWEMARC), University of Gothenburg, Box 463, 405 30, Gothenburg, Sweden; 3 Blue Food Center, University of Gothenburg, Box 463, 405 30, Gothenburg, Sweden; 4 Department of Civil and Environmental Engineering, Graduate School of Advanced Science and Engineering, Hiroshima University, 1–4–1, Kagamiyama, Higashihiroshima, Hiroshima 739–8527, Japan; 5 Dairy Ecosystem Research & Development Center, Hiroshima University, 2–2965, Kagamiyama, Higashihiroshima, Hiroshima, 739–0046, Japan

**Keywords:** Anammox, *Candidatus* Scalindua sp., nitrate tolerance, wastewater treatment, removal efficiency

## Abstract

To investigate the NO_3_^–^ tolerance of *Candidatus* Scalindua sp., a continuous reactor was gradually exposed to increasing NO_3_^–^ concentrations up to 3,200 mg N L^–1^. High NH_4_^+^ and NO_2_^–^ removal efficiencies were maintained up to 2,600 mg N L^–1^, above which performance declined and *Ca.* Scalindua relative abundance decreased to 0.8%. After one year of recovery, removal efficiencies exceeded 97%, whereas *Ca.* Scalindua relative abundance only reached 6.5%. EC_50_ values for NH_4_^+^ and NO_2_^–^ were both 3,000 mg N L^–1^. We demonstrated that our enriched *Ca.* Scalindua population tolerated NO_3_^–^ up to 2,600 mg N L^–1^, far exceeding the levels typically encountered in most human-derived wastewaters.

The anammox (anaerobic ammonium oxidation) process is a chemoautotrophic nitrogen removal process by which ammonium (NH_4_^+^), the electron donor, is transformed into dinitrogen gas (N_2_) using nitrite (NO_2_^–^) as the electron acceptor (Strous *et al.*, 1999; Kartal *et al.*, 2012). This process is performed by anaerobic ammonium-oxidizing bacteria (AnAOB) belonging to the phylum *Planctomycetes* and the order *Candidatus* Brocadiales. Among these, *Candidatus* Scalindua is the only known marine anammox genus (Zhang and Okabe, 2020). Due to its limited need for external carbon and its low emission of greenhouse gases, this process has been recognized as a cost-effective and environmentally friendly nitrogen removal method from wastewater, including municipal (Zhao *et al.*, 2022), industrial (Li *et al.*, 2018), and, more recently, aquaculture origins (Micolucci *et al.*, 2023).

However, AnAOB are sensitive to changes in environmental conditions, including a suboptimal concentration of nitrogen waste originating from the environment or locally produced by cohabitating organisms in the biofilm. Anammox bacteria are generally not present as a monoculture, they cohabit with other bacteria, such as ammonia-oxidizing bacteria (AOB), nitrite-oxidizing bacteria (NOB), and heterotrophic bacteria (HB). Cooperation and competition among these microorganisms are critical to the stability and performance of the anammox process within a reactor (Li *et al.*, 2018). Since anammox bacteria have a slow growing rate (Awata *et al.*, 2013), any changes in key nitrogen parameters, including nitrate (NO_3_^–^) produced by NOB (within the granule or upstream of the reactor) or already present in industrial wastewater, may impair its normal functioning and change its abundance withing the granule (Roques *et al.*, 2021; Roques *et al.*, 2024). Therefore, the specific requirements for optimal wastewater treatment under all system conditions, including NO_3_^–^ using *Ca.* Scalindua, must be identified. We previously investigated the impact of NO_3_^–^ levels typically found in marine recirculating aquaculture systems (RAS), showing that the removal efficiencies for both NH_4_^+^ and NO_2_^–^ remained high and constant (above 95%) at the highest concentration tested (400 mg N L^–1^) (Roques *et al.*, 2024). However, we also observed a decline in the relative abundance of our *Ca.* Scalindua population in favor of an expansion of HB potentially capable of NO_3_^–^ reduction (Roques *et al.*, 2024).

Fish farmers typically initiate actions when NO_3_^–^ levels reach approximately 100 mg N L^–1^ to avoid health and welfare impairments (Banerjee *et al.*, 2023; Roques *et al.*, 2024); however, concentrations approaching 1,000 mg N L^–1^ have been reported in poorly controlled RAS (Van Rijn, 2013). Similar concentrations have been found in industrial wastewater from cellophane, fertilizer, pectin, metal finishing, and explosives industries, reaching up to 3,000 mg N L^–1^ for the latter (Fernández-Nava *et al.*, 2008; Cyplik *et al.*, 2012). Therefore, the aim of the present study was to investigate the maximum tolerance of *Ca.* Scalindua to NO_3_^–^ with a view to its application to wastewater treatment systems in which NO_3_^–^ levels may be elevated and, thus, a limiting factor for the anammox process.

The granular biomass (6.5 g wet weight) from a *Ca.* Scalindua-dominated enrichment reactor cultivated at the University of Gothenburg (Sweden) since 2019 (Micolucci *et al.*, 2023), which was originally derived from a long-term up-flow anammox enrichment reactor at Hiroshima University (Japan) (Kindaichi *et al.*, 2011), was used as the inoculum for a new reactor, operated for 956 days in five experimental phases (Table 1, Fig. 1).

During Phase 1 (Stabilization, days 0–52), the reactor was fed with the same standard synthetic marine wastewater feed supplemented with NH_4_^+^, NO_2_^–^, inorganic carbon (KHCO_3_), and a mineral and trace elements mix (van de Graaf *et al.*, 1996; Roques *et al.*, 2021). During Phase 2 (days 53–170), the reactor was exposed to gradually increasing concentrations of NO_3_^–^, supplied as sodium nitrate (NaNO_3_), starting from 200 mg N L^–1^ and reaching 3,200 mg N L^–1^ on day 141. Following a reduction in removal efficiencies after 30 days at 3,200 mg N L^–1^, NO_3_^–^ concentrations were decreased and subsequently maintained at 1,600 mg N L^–1^ for recovery during Phase 3 (days 171–368). During Phase 4 (days 369–589), the reactor was exposed to gradually increasing concentrations of NO_3_^–^, starting from 2,000 mg N L^–1^ and reaching 3,000 mg N L^–1^ on day 513. During Phase 5 (Recovery, days 590–956), the reactor was operated without the addition of NO_3_^–^, while NH_4_^+^ (56 mg N L^–1^), NO_2_^–^ (68 mg N L^–1^), and KHCO_3_ (1,500 mg L^–1^) were increased. All other environmental parameters were kept unchanged (Table 1, Fig. S1).

During Phase 1, NH_4_^+^ and NO_2_^–^ removal efficiencies reached 75–100% after 18 days (Fig. 1), while NO_3_^–^ exhibited a high negative removal efficiency (*i.e.*, it was produced) of *ca.* –600%. *Ca.* Scalindua was also the most dominant species in the granule (28.3%, Fig. 2), indicating the successful establishment of the anammox process (Kindaichi *et al.*, 2011). During Phase 2, high NH_4_^+^ (87.7±7.8%) and NO_2_^–^ (95.4±4.1%) removal efficiencies were maintained until NO_3_^–^ concentrations reached 3,200 mg N L^–1^, at which point the reactor crashed (Fig. 1 and S2). During this phase, the relative abundance of *Ca.* Scalindua decreased to 12.6% when chronically exposed to 1,600 mg N L^–1^ (day 138) and further declined to 0.8% after 30 days at 3,200 mg N L^–1^ (day 168). NO_3_^–^ removal efficiencies during these phases were 2.2±5.8 and 2.4±3.7%, respectively, indicating net NO_3_^–^ removal, representing only‍ ‍a small fraction of the total NO_3_^–^ pool under these conditions. In parallel, there were increases in *Firmicutes*‍ ‍(*Bacillaceae*), *Alphaproteobacteria* (mainly *Rhodobacteraceae*), and *Bacteroidota* (*Flavobacteriaceae* and *Saprospiraceae*), which replaced *Ca.* Scalindua and *Actinobacteria* (order *Actinomarinales*) (Fig. 2). Several of these enriched taxa include lineages reported to possess NO_3_^–^-reducing or denitrifying capabilities under anoxic conditions (Parkes *et al.*, 2014; Mandic-Mulec *et al.*, 2016; Shan *et al.*, 2024). However, since denitrification intermediates and functional genes were measured in this study, it was not possible to directly quantify the contribution of denitrification.

NH_4_^+^ and NO_2_^–^ removal efficiencies were restored to >75% after three months under 1,600 mg N L^–1^ during Phase 3 (Fig. 1). *Ca.* Scalindua relative abundance increased to 13.1%, while the other groups, with the exception of *Melioribacteraceas*, returned to pre- NO_3_^–^ exposure levels (Fig. 2).

In Phase 4, the reactor started to become unstable again from exposure to 2,600 mg N L^–1^ onward, where NH_4_^+^ and NO_2_^–^ removal efficiencies fell below 50% (Fig. 1). Consequently, the relative abundance of *Ca.* Scalindua decreased to 5.3% (Fig. 2). Net NO_3_^–^ removal efficiencies were negligible and highly variable during this phase. Following the removal of NO_3_^–^ during Phase 5, high NH_4_^+^ (97.2±2.6%) and NO_2_^–^ (99.7±0.7%) removal efficiencies were achieved again (Fig. 1), and were accompanied by large net NO_3_^–^ production (–920±265%). This stoichiometric pattern is consistent with the reestablishment of anammox as the dominant nitrogen transformation pathway in the reactor (Kindaichi *et al.*, 2011).

Despite NH_4_^+^ and NO_2_^–^ being the sole nitrogen sources supplied in Phase 5, *Ca.* Scalindua relative abundance remained moderate (6.5%) rather than being further enriched. *Firmicutes* quasi disappeared, while the percentages of some *Proteobacteria*, *Bacteroidota* (including *Flavobacteriaceae*), *Chloroflexi*, and *Planctomycetes* (excluding *Ca.* Scalindua) were higher than the pre- NO_3_^–^ exposure phase (Fig. 2). The inoculum originated from a long-term enrichment reactor in which *Ca.* Scalindua was dominant but coexisted with other taxa. Throughout operation, all major bacterial groups were already present from the start, and no new taxa emerged; their relative abundance shifted in response to operational conditions. Strong biomass retention provided by the non-woven fabric carrier promoted attached-growth biofilm formation, minimizing washout and allowing multiple functional guilds to persist within the reactor (Ren *et al.*, 2018).

Importantly, no alternative anammox lineage increased, indicating that fluctuations reflected changes in the abundance of *Ca.* Scalindua rather than clade replacement. The marked decline in *Ca.* Scalindua during high NO_3_^–^ exposure followed by partial recovery may reflect a bottleneck event after which a more tolerant fraction of the population persisted. Given the slow growth of anammox bacteria (Awata *et al.*, 2013), full return to initial dominance may be limited. However, strain-level selection cannot be resolved from our 16S rRNA gene data.

Regarding non-anammox community changes, *Firmicutes* (*Bacillaceae*) are mostly fast-growing generalist heterotrophs that are capable of fermentation and NO_3_^–^ reduction or denitrification and may form spores, making them relatively resistant to environmental stress (Parkes *et al.*, 2014; Mandic-Mulec *et al.*, 2016). They are known to thrive during early community shifts under perturbations, such as that observed during Phase 2, before disappearing once conditions become more stable or favorable for other bacterial groups thriving on high NO_3_^–^. A similar pattern was reported by Zhang *et al.* (2022) where 25–100 mg N L^–1^ led to a decrease in the abundance of *Firmicutes* in groundwater. This is consistent with our previous study where *Firmicutes* also almost disappeared after a chronic exposure to 400 mg N L^–1^ (Roques *et al.*, 2024). The anaerobic versatile *Alphaproteobacteria* (HB) may have benefited from the higher NO_3_^–^ sensitivity of *Firmicutes* to outcompete this phylum in the long term (Qu *et al.*, 2021; Roques *et al.*, 2024). *Bacteroidota*, *Flavobacteriaceae*, *Saprospiraceae*, and, to a lesser extent, photosynthetic *Chloroflexi* likely served as organic matter recyclers, scavenging dead *Firmicutes* or other bacteria to flourish after chronic exposure to high NO_3_^–^ levels (Yang *et al.*, 2018; Roques *et al.*, 2024).

Based on the removal efficiencies of both NH_4_^+^ and NO_2_^–^, concentration–response curves for NO_3_^–^ were generated (Fig. 3). Half maximal effective concentration values (EC_50_) and their 95% confidence intervals were 3,000 (2,930–3,090) and 3,000 (2,910–3,110) mg N L^–1^ for NH_4_^+^ and NO_2_^–^, respectively, indicating an exceptionally high tolerance of *Ca.* Scalindua to elevated NO_3_^–^ concentrations.

Although we noted a reduction in the relative abundance of our population of *Ca.* Scalindua in the granule (from 28.3% at the beginning of the experiment to 5.3% after 19 months of exposure to fluctuating high NO_3_^–^ concentrations and to 6.5% after one year of recovery), high NH_4_^+^ and NO_2_^–^ removal efficiencies coupled with net anammox-consistent NO_3_^–^ production and fluorescence *in situ* hybridization (FISH) observations (Fig. S3) indicated the presence of a functioning, stable population of *Ca.* Scalindua throughout the experiment. Based on our results, we conclude that the enriched *Ca.* Scalindua population investigated in this study is highly tolerant to high levels of NO_3_^–^ up to concentrations of 2,600 mg N L^–1^, far exceeding the levels typically encountered in most human-derived wastewaters. Collectively, these results suggest that marine anammox systems enriched with *Ca.* Scalindua are broadly applicable for nitrogen removal from a wide variety of wastewater sources containing high NO_3_^–^ levels. However, the precise cellular and molecular mechanisms underlying NO_3_^–^-associated inhibition and acclimation remain unclear and warrant further investigation.

## Acknowledgements

The present study was conducted within the frame of the MIRAI project and was supported by FORMAS (2020-00867), Kungl. Skogs-och Lantbruksakademien (GFS2024-0148), STINT (mobility grant for internationalization, MG2019-8483) in Sweden; JSPS KAKENHI (JP23KJ1642, JP24KK0197), JSPS Bilateral Program (JPJSBP120259928) and FY2025 JSPS Invitational Fellowship for Research in Japan (S25124) in Japan. The authors thank Lise Brault, Linda Frank Hasselberg, and Thanh Nguyen Duc at the University of Gothenburg for their technical assistance.

### Conflicts of interest

The authors declare that there are no conflicts of interest.

## Citation

Roques, J. A. C., Fujii, N., Unegbu, E., Marqué, A., Johansson, E., Yamamoto, K., et al. (2026) Performance of Marine Anammox *Candidatus* Scalindua sp. under High Nitrate Conditions in a Biofilm Reactor. *Microbes Environ ***41**: ME25094.

https://doi.org/10.1264/jsme2.ME25094

## Supplementary Material

Supplementary Material

## Figures and Tables

**Fig. 1.**
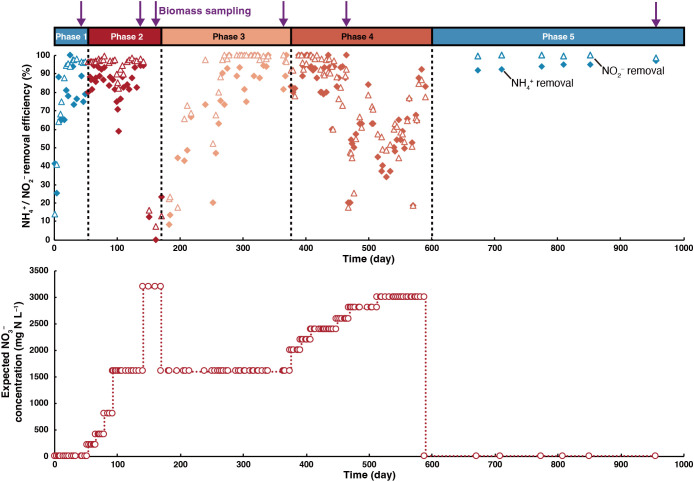
Anammox performance in the reactor. (**A**) NH_4_^+^ (closed diamonds) and NO_2_^–^ (open triangles) removal efficiencies (%). Purple arrows indicate biomass sampling on days 38, 138, 167, 340, 469, and 956. (**B**) Expected NO_3_^–^ concentrations (dotted lines and open circles).

**Fig. 2.**
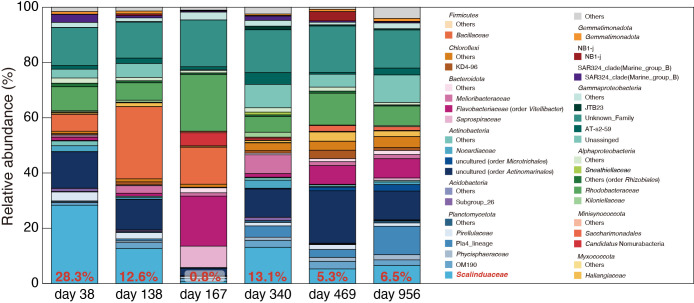
Microbial community composition at the end of Phase 1 (day 38), during Phase 2 after 48 days of exposure to 1,600 mg N L^–1^ (day 138), after 30 days of exposure to 3,200 mg N L^–1^ (day 167), at the end of Phase 3 (after 5.5 months of constant exposure to 1,600 mg N L^–1^, day 340), in the middle of Phase 4 (2,800 mg N L^–1^ day 469), and at the end of Phase 5 (0 mg N L^–1^, recovery, day 956). An anal­ysis based on 16S rRNA gene amplicon sequencing. Red percentages correspond to the relative abundance of the marine anammox *Ca.* Scalindua.

**Fig. 3.**
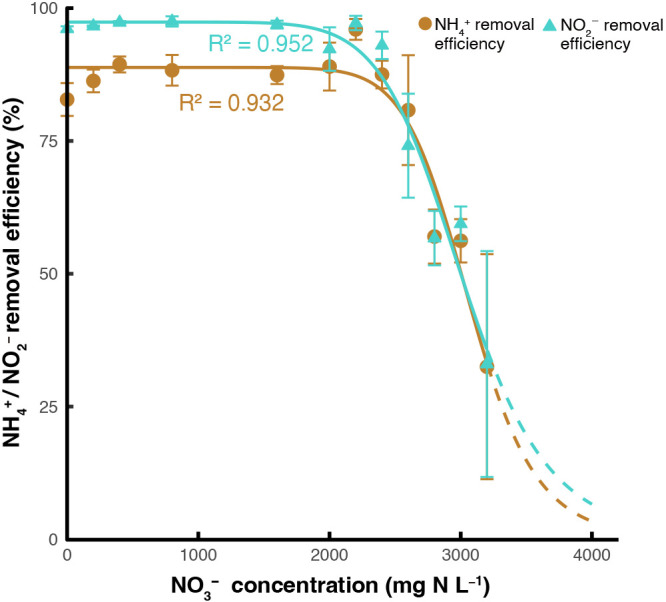
Concentration-response curves for NH_4_^+^ and NO_2_^–^ as a function to ambient NO_3_^–^ concentrations (in mg N L^–1^). The curves were calculated using data obtained from day 18 (end of the stabilization period in Phase 1) to day 589 (end of Phase 4). EC_50_ values (95% confidence intervals) were 3,000 (2,930–3,090) and 3,000 (2,910–3,110) mg N L^–1^ for NH_4_^+^ and NO_2_^–^, respectively. The fitted models were y=88.8*(1+exp[12.1*{log x–8.0}])^–1^ (R^2^=0.932) for NH_4_^+^ and y=97.4 * (1+exp[9.3*{log x–8.0}])^–1^ (R^2^=0.952) for NO_2_^–^.

**Table 1.** Operational conditions of the column reactor in three experimental phases. Values represent the average (±standard deviation). *HRT: hydraulic retention time*

Phase	Period (day)	[NO_3_^–^ N] (mg L^–1^)	HRT (h)	pH influent	pH effluent	Nitrogen loading rate (g TN L^–1^ day^–1^)	Nitrogen removal rate (g TN L^–1^ day^–1^)	NH_4_^+^ and NO_2_^–^ loading rate (g TN L^–1^ day^–1^)	NH_4_^+^ and NO_2_^–^ removal rate (g TN L^–1^ day^–1^)
1	0–52	0	4.9 (0.4)	7.13 (0.13)	7.47 (0.15)	0.30±0.04	0.21±0.07	0.30±0.04	0.22±0.06
2	53–170	200 → 3,200	5.3 (1.0)	7.19 (0.23)	7.55 (0.21)	6.06±4.29	0.28±0.57	0.28±0.03	0.23±0.07
3	171–368	1,600	6.3 (1.8)	7.34 (0.21)	7.65 (0.18)	6.59±1.37	0.45±0.40	0.26±0.05	0.21±0.07
4	369–589	2,000 → 3,000	4.7 (0.5)	7.33 (0.32)	7.50 (0.29)	13.69±2.58	0.23±0.86	0.35±0.05	0.25±0.07
5	589–956	0	3.9 (0.6)	7.65 (0.39)	7.78 (0.47)	0.77±0.14	0.71±0.15	0.77±0.14	0.75±0.14
