# Supplementary material for: Performance of Marine Anammox Candidatus Scalindua sp. under High Nitrate Conditions in a Biofilm Reactor
Source: Microbes Environ. 2026 May 27;41(2):ME25094. doi: 10.1264/jsme2.ME25094 (PMC13293702; doi:10.1264/jsme2.ME25094)
Supplement: Supplementary file 1 — Supplementary Material [file 41_25094_s1.pdf]

Performance of the marine anammox bacteria *Candidatus* Scalindua under high nitrate conditions

– Supporting information –

Jonathan A.C. Roques[1,2,3,†,*], Naoki Fujii[4,†], Ebuka Unegbu[1], Amélie Marqué[1], Emma Johansson[1], Kohei Yamamoto[4], Haruhi Iida[4], Tomonori Kindaichi[4,*]

[1] Department of Biological and Environmental Sciences, University of Gothenburg, Box 463, 405 30, Gothenburg, Sweden
[2] Swedish Mariculture Research Center (SWEMARC), University of Gothenburg, Box 463, 405 30, Gothenburg, Sweden
[3] Blue Food Center, University of Gothenburg, Box 463, 405 30, Gothenburg, Sweden
[4] Department of Civil and Environmental Engineering, Graduate School of Advanced Science and Engineering, Hiroshima University,1-4-1, Kagamiyama, Higashihiroshima, Hiroshima 739-8527, Japan

[†] These authors contributed equally to this work

[*] Co-corresponding authors:
Jonathan A.C. Roques, jonathan.roques@bioenv.gu.se,
Tomonori Kindaichi, E-mail: tomokin@hiroshima-u.ac.jp

Materials and Methods

Reactor operation

Ca. Scalindua granules (ca. 5 g wet weight) from an up-flow column anammox stock culture at Hiroshima University (Higashihiroshima, Japan), in operation for more than 13 years (Kindaichi *et al.,* 2011a, Kindaichi *et al.,* 2011b, Mojiri *et al.,* 2018), were used to inoculate a glass a glass column reactor at the University of Gothenburg (Sweden) in 2019 (φ 50 mm; volume, 290 cm^3, KF-50, AS ONE, Tokyo, Japan), using a new non-woven fabric sheet (Japan Vilene, Tokyo, Japan). The reactor was fed with synthetic marine wastewater (salinity: 29 ‰, Aquaforest, Brzesko, Poland) supplemented with nitrogen; ammonium (NH_4^+, 28 mg-N L^{-1}) and nitrite (NO_2^-, 34 mg-N L^{-1}), inorganic carbon in the form of potassium bicarbonate ($KHCO_3$, 1000 mg L^{-1}), mineral and trace elements mix, as described earlier (van de Graaf *et al.,* 1996, Micolucci *et al.,* 2023, Roques *et al.,* 2024). After adding all the different chemicals, the wastewater feed was flushed with dinitrogen gas (N_2) for at least 30 minutes to achieve a concentration of dissolved oxygen (O_2) below 0.5 mg L^{-1}, and the pH was adjusted to ca. 7.0–7.5 with a solution of glacial sulfuric acid (H_2SO_4) (van de Graaf *et al.,* 1996). The influents were continuously introduced into the reactor using a peristaltic pump (Masterflex L/S Economy Drive, Cole-Parmer Instruments, Vernon Hills, USA).

On January 10th, 2023 (ca. 1230 days after the initial inoculation), 6.5 g (wet weight) of biomass from the stock reactor were inoculated into a new reactor of the same volume as the original inoculum, using a new nonwoven fabric sheet of the same kind. The reactor used in this study was operated for 956 days in five experimental phases to evaluate the tolerance of *Ca.* Scalindua to nitrate (NO_3^-, Table 1).

Analytical methods

NH_4^+, NO_2^-, and NO_3^- concentrations in the influent and effluent are presented in Figure S1. The removal efficiencies for individual nitrogen compounds (Figure 1), the total nitrogen (TN) loading and removal rates and the HRT (Table 1, Figure S2) were calculated according to the following:

Removal efficiency (Nx, %) = [[Influent Nx (g-N L^{-1})] - [Effluent Nx (g-N L^{-1})]] / [Influent Nx (g-N L^{-1})] * 100

Where Nx is the desired nitrogen compound among NH_4^+, NO_2^-, or NO_3^-

Loading rate (g-TN L^{-1} day^{-1}) = [[Influent NX (g-N L^{-1})]] * [Influent volumetric flow rate (L day^{-1})] / [Volume of the tank (L)]

Removal rate (g-TN L^{-1} day^{-1}) = [[Influent NX (g-N L^{-1})] - [Effluent NX (g-N L^{-1})]] * [Influent volumetric flow rate (L day^{-1})] / [Volume of the tank (L)]

Where NX is the sum of NH_4^+, NO_2^-, and NO_3^-

HRT (h) = Volume of the reactor (L) / Influent volumetric flow rate (L h^{-1})

Salinity, O_2 levels and temperature were determined using a conductivity meter portable Multimeter pHenomenal MU 6100 H (VWR international, Radnor, USA). NH_4^+ concentrations were determined using the powder pillow methods (salicylate method, 8155, Hach-Lange, Dusseldorf, Germany), NO_2^- concentrations were determined using the LCK341 kit (Hach-Lange, Dusseldorf, Germany) and the DR-2800 spectrophotometer (Hach-Lange, Dusseldorf, Germany) (Roques *et al.,* 2024). NO_3^- concentrations were determined using ion-exchange chromatography (HPLC 20A; Shimadzu, Kyoto, Japan) with a Shodex Asahipak NH2P-50 4D anion column (Showa Denko, Tokyo, Japan) and UV-VIS detector (SPD-20AV, Shimadzu) after filtration of samples through 0.2-μm pore-size PTFE membranes (Advantec, Tokyo, Japan) (Mojiri *et al.,* 2020).

Concentration-effect curves

Concentration–response curves for NH_4^+ and NO_2^- removal efficiencies were fitted using a log-logistic model following Seefeldt *et al.,* (1995). The model was adjusted by nonlinear least-squares regression using the nlsLM function in the minpack.lm package implemented in R (R-Core-Team 2023). Data obtained between day 18 and day 589 were used for model fitting. EC_{50} values and their 95% confidence intervals were calculated from the fitted parameters (Figure 3).

16S rRNA gene amplicon sequence analysis

To determine the potential changes in the microbial community composition during the different phases, biomass samples for the amplicon sequencing were collected six times from the reactor: at the end of the stabilization phase (day 38); before and after the marked change in removal efficiency observed in Phase 2 (days 138 and 167); when the removal efficiency had fully recovered in Phase 3 (day 340); at the midpoint of Phase 4 (day 469); and on the final day of the experiment (day 956) (Figure 2). DNA was extracted using a FastDNA SPIN kit for soil (MP Biomedicals, Santa Ana, USA). PCR amplification of the bacterial 16S rRNA gene was performed with a primer set for amplification of the V3-V4 region as follows: 341F (5′-CCTACGGGNGGCWGCAG-3′) and 805R (5′-GGACTACHVGGGTATCTAATCC-3′). The details of PCR amplification were as described previously (Shoiful *et al.,* 2020). PCR products were purified using the Agencourt AMPure XP system (Beckman Coulter, Brea, USA) according to manufacturer instructions. Purified DNA was sequenced using a MiSeq platform with a MiSeq reagent kit (v.3; Illumina, San Diego, USA).

Obtained sequences were trimmed and assembled as described previously (Awata *et al.,* 2021). Sequence data were analyzed using QIIME 2 Amplicon 2025.10 distribution (Bolyen *et al.,* 2019). Amplicon Sequence Variants (ASVs) were assigned with the SILVA 138.1 database (Quast *et al.,* 2012). ASVs that accounted for over 1% of the total reads were used for bar-plots representation (Figure 2). The sequence data in the present study was deposited in the DNA Data Bank of Japan (DDBJ) database under the DDBJ/EMBL/GenBank under the Bioproject ID PRJDB10627.

Fluorescence in situ hybridization (FISH)

Biomass samples were collected from the up-flow column reactor at the same time-points as for the microbial community analysis (days 38, 138, 167, 340, 469 and 956). Pictures of the end of the

stabilization phase (day 38), at the end of Phase 4 (day 340) and on the last day of the recovery phase (day 956) are presented in Figure S3. Sample fixation and the following FISH procedure were performed as previously described (Awata *et al.,* 2013). Probes were labelled at the 5' end with either Alexa Fluor 555 or Alexa Fluor 647 (Table S1). Hybridized samples were observed with an AxioImager Z2 epifluorescence microscope with a 100 W HBO lamp (Carl Zeiss, Oberkochen, Germany). Images were obtained using an AxioCam 712 mono camera and AxioVision software, version 4.5 (Carl Zeiss, Oberkochen, Germany).

Table S1. Oligonucleotide probes used for FISH analysis in this study.

Probe	Target group	Sequence (5' to 3')	FA (%)[1]	Reference
EUB338	Most Bacteria	GCT GCC TCC CGT AGG AGT	0–50	Amann *et al.,* (1990)
EUB338II	*Planctomycetales*	GCA GCC ACC CGT AGG TGT	0–50	Daims *et al.,* (1999)
EUB338III	*Verrucomicrobiales*	GCT GCC ACC CGT AGG TGT	0–50	Daims *et al.,* (1999)
EUB338IV	Bacteria lineages not covered by EUB338, EUB338II, and EUB338III	GCA GCC TCC CGT AGG AGT	0–50	Schmid *et al.,* (2005)
Sca1129b	*Ca.* Scalindua sp.	TAC TCG GCA TTA CCC GAT	15	Kindaichi *et al.,* (2011a)

[1] FA: formamide (% v/v) used in the hybridization buffer

References

Amann, R.I., Binder, B.J., Olson, R.J., Chisholm, S.W., Devereux, R. and Stahl, D. (1990). "Combination of 16S rRNA-targeted oligonucleotide probes with flow cytometry for analyzing mixed microbial populations." Appl. Environ. Microbiol. 56(6): 1919-1925

Awata, T., Goto, Y., Kuratsuka, H., Aoi, Y., Ozaki, N., Ohashi, A. and Kindaichi, T. (2021). "Reactor performance and microbial community structure of single-stage partial nitritation anammox membrane bioreactors inoculated with Brocadia and Scalindua enrichment cultures." Biochem. Eng. J. 170: 107991

Awata, T., Oshiki, M., Kindaichi, T., Ozaki, N., Ohashi, A. and Okabe, S. (2013). "Physiological characterization of an anaerobic ammonium-oxidizing bacterium belonging to the "Candidatus Scalindua" group." Appl. Environ. Microbiol. 79(13): 4145-4148

Bolyen, E., Rideout, J.R., Dillon, M.R., Bokulich, N.A., Abnet, C.C., Al-Ghalith, G.A.*, et al.* (2019). "Reproducible, interactive, scalable and extensible microbiome data science using QIIME 2." Nat. Biotechnol. 37(8): 852-857

Daims, H., Brühl, A., Amann, R., Schleifer, K.-H. and Wagner, M. (1999). "The domain-specific probe EUB338 is insufficient for the detection of all Bacteria: development and evaluation of a more comprehensive probe set." Syst. Appl. Microbiol. 22(3): 434-444

Kindaichi, T., Awata, T., Suzuki, Y., Tanabe, K., Hatamoto, M., Ozaki, N. and Ohashi, A. (2011a). "Enrichment using an up-flow column reactor and community structure of marine anammox bacteria from coastal sediment." Microbes Environ. 26(1): 67-73

Kindaichi, T., Awata, T., Tanabe, K., Ozaki, N. and Ohashi, A. (2011b). "Enrichment of marine anammox bacteria in Hiroshima Bay sediments." Water Sci. Technol. 63(5): 964-969

Micolucci, F., Roques, J.A.C., Ziccardi, G.S., Fujii, N., Sundell, K. and Kindaichi, T. (2023). "*Candidatus* Scalindua, a Biological Solution to Treat Saline Recirculating Aquaculture System Wastewater." Processes 11(3): 690

Mojiri, A., Nishimoto, K., Awata, T., Aoi, Y., Ozaki, N., Ohashi, A. and Kindaichi, T. (2018). "Effects of salts on the activity and growth of "Candidatus Scalindua sp.", a marine Anammox bacterium." Microbes Environ. 33(3): 336-339

Mojiri, A., Ohashi, A., Ozaki, N., Aoi, Y. and Kindaichi, T. (2020). "Integrated anammox-biochar in synthetic wastewater treatment: performance and optimization by artificial neural network." J. Cleaner Prod. 243: 118638

Quast, C., Pruesse, E., Yilmaz, P., Gerken, J., Schweer, T., Yarza, P., *et al.* (2012). "The SILVA ribosomal RNA gene database project: improved data processing and web-based tools." Nucleic Acids Res. 41(D1): D590-D596

R-Core-Team (2023). "R: A language and environment for statistical computing. R Foundation for Statistical Computing, Vienna, Austria." https://www.r-project.org/

Roques, J.A.C., Unegbu, E., Fujii, N., Marqué, A., Micolucci, F., Sundell, K.S. and Kindaichi, T. (2024). "Tolerance of the Marine Anammox *Candidatus* Scalindua to High Nitrate Concentrations: Implications for Recirculating Aquaculture Systems." Water 16(24): 3705

Schmid, M.C., Maas, B., Dapena, A., van de Pas-Schoonen, K., van de Vossenberg, J., Kartal, B., *et al.* (2005). "Biomarkers for in situ detection of anaerobic ammonium-oxidizing (anammox) bacteria." Appl. Environ. Microbiol. 71(4): 1677-1684

Seefeldt, S.S., Jensen, J.E. and Fuerst, E.P. (1995). "Log-logistic analysis of herbicide dose-response relationships." Weed Technol. 9(2): 218-227

Shoiful, A., Kambara, H., Cao, L.T.T., Matsushita, S., Kindaichi, T., Aoi, Y., *et al.* (2020). "Mn (II) oxidation and manganese-oxide reduction on the decolorization of an azo dye." Int. Biodeterior. Biodegrad. 146: 104820

van de Graaf, A.A., de Bruijn, P., Robertson, L.A., Jetten, M.S. and Kuenen, J.G. (1996). "Autotrophic growth of anaerobic ammonium-oxidizing micro-organisms in a fluidized bed reactor." Microbiology 142(8): 2187-2196

Supplementary Figures

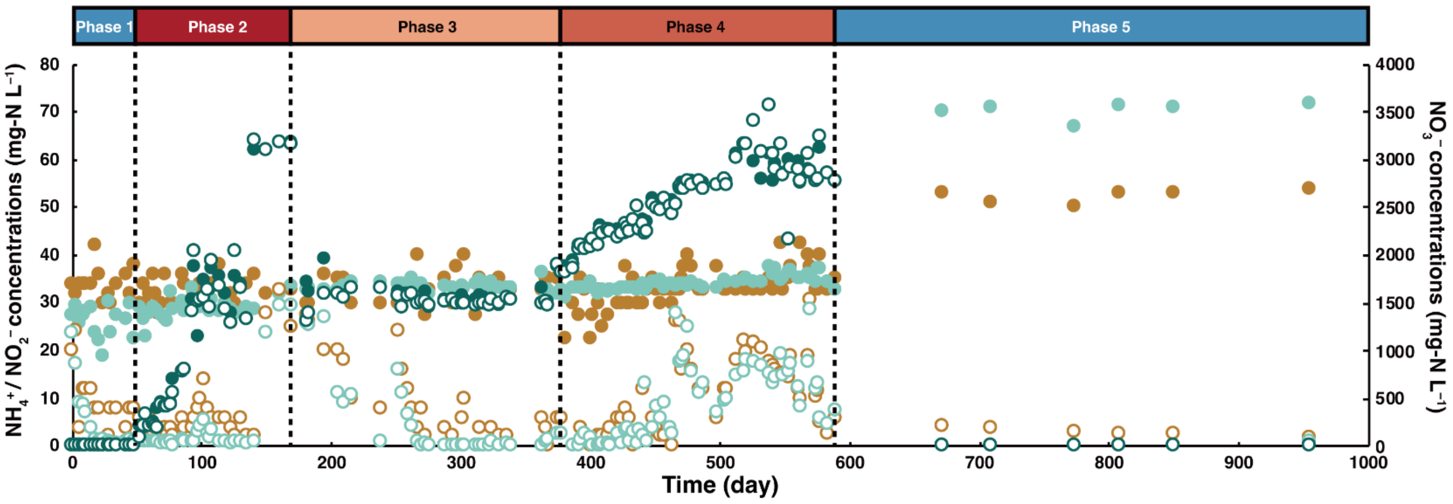

Figure S1. Concentrations of NH_4^+ (brown), NO_2^- (light green) and NO_3^- (dark green) in the influent (filled circles) and effluent (open circles) during the five experimental phases (mg-N L^{-1}).

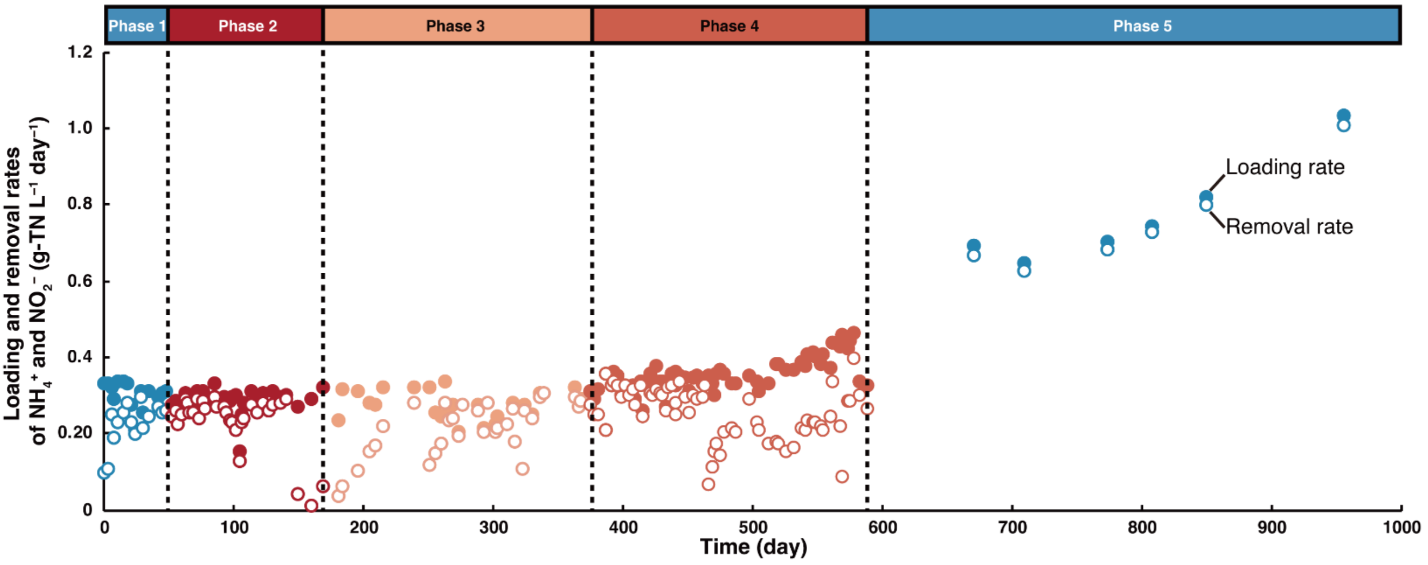

Figure S2. Nitrogen loading and removal rates (filled and open circles, respectively) during the five experimental phases (g-TN L^{-1} day^{-1}).

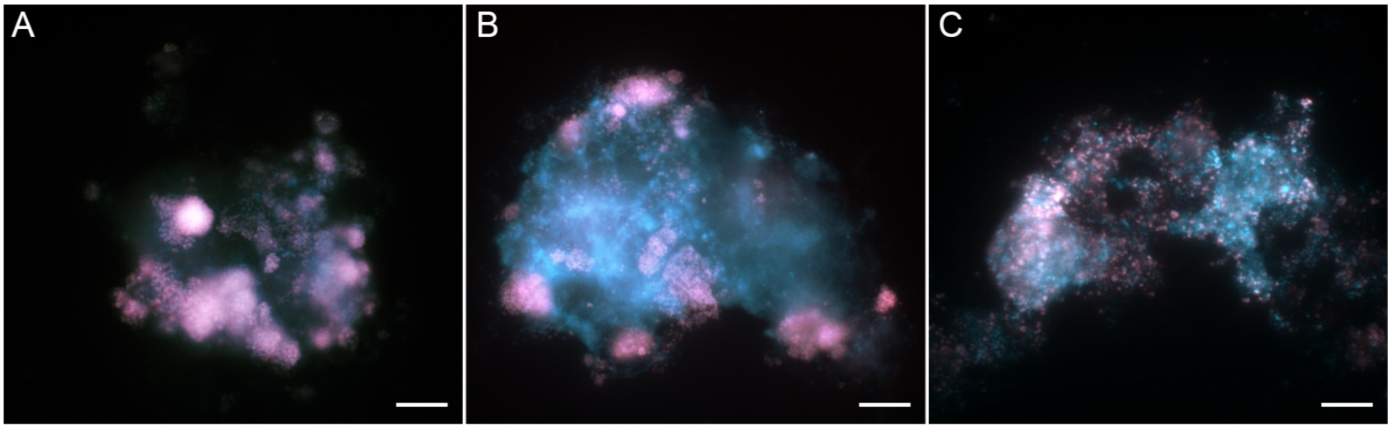

Figure S3. FISH micrographs of biomass collected from the reactor on days 38 (A), day 340 (B) and 956 (C), FISH analysis utilized the Alexa Fluor 555 and 647 labelled probes. *Ca.* Scalindua appears magenta and other bacteria appear blue. Scale bars indicate 10 μm.
